# On the Validity of Adipogenic Cell Lines as Model Systems for Browning Processes: In Authentic Brown, Brite/Beige, and White Preadipocytes, There is No Cell-Autonomous Thermogenic Recruitment by Green Tea Compounds

**DOI:** 10.3389/fnut.2021.715859

**Published:** 2021-08-17

**Authors:** Rosemari Otton, Natasa Petrovic, Barbara Cannon, Jan Nedergaard

**Affiliations:** ^1^Interdisciplinary Post-Graduate Programme in Health Sciences, Cruzeiro do Sul University, São Paulo, Brazil; ^2^Department of Molecular Biosciences, The Wenner-Gren Institute, Stockholm University, Stockholm, Sweden

**Keywords:** thermogenesis, adipogenesis, polyphenols, PPAR γ, green tea (*Camellia sinensis* L.)

## Abstract

The potential ability of nutritional compounds to induce or enhance the browning of adipocytes has attracted large interest as a workable means of combatting the obesity epidemic. Green tea compounds are discussed as such inducers of an enhanced thermogenic capacity and activity. However, the cell-autonomous effects of green tea compounds on adipocytes have until now only been demonstrated in adipogenic cell lines (3T3-L1 and 3T3-F442A), i.e., cells of undefined tissue lineage. In this study, we examine the ability of green tea compounds to cell-autonomously induce thermogenic recruitment in authentic brown and brite/beige adipocytes *in vitro*. In primary brown adipocytes, the green tea compounds suppressed basal UCP1 gene expression, and there was no positive interaction between the compounds and adrenergic stimulation. In white adipocytes, green tea compounds decreased both basal and norepinephrine-induced UCP1 mRNA levels, and this was associated with the suppression of cell differentiation, indicated by reduced lipogenic gene expression and lipid accumulation. A lack of interaction between rosiglitazone and green tea compounds suggests that the green tea compounds do not directly interact with the PPARγ pathway. We conclude that there is a negative effect of the green tea compounds on basal UCP1 gene expression, in both brown and white primary adipocytes, in contrast to the positive effects earlier reported from studies in adipogenic cell lines. We posit that the epigenetic status of the adipogenic cell lines is fundamentally different from that of genuine brown and white adipocytes, reflected, e.g., in several-thousand-fold differences in UCP1 gene expression levels. Thus, results obtained with adipogenic cell lines cannot unreservedly be extrapolated as being relevant for authentic effects in brown and white adipocytes. We suggest that this conclusion can be of general concern for studies attempting to establish physiologically relevant cell-autonomous effects.

## Introduction

A potential means to ameliorate the present obesity pandemic would be to increase energy expenditure. In this context, the possibility to recruit the thermogenic capacity of brown adipose tissue (BAT) and possibly induce “browning” of certain white adipose tissue depots has attracted attention (1), especially after it has become accepted that adult humans can possess active BAT (2, 3).

A conceptually attractive means of enhancing adipose tissue thermogenesis is through the use of nutraceuticals. One much-studied nutraceutical is green tea. Particularly, since the observation of Dulloo et al. that the ingestion of green tea compounds may elicit a metabolically significant (4%) increase in 24-h energy expenditure in adult humans (4), green tea itself, green tea extracts (GTEs), or purified polyphenols (particularly catechins) from the *Camellia sinensis* plant (collectively referred to as “green tea compounds”) have been studied for their possible thermogenic effects in humans. A meta-analysis of five studies concluded that green tea compounds increase energy expenditure by about 5% (but this may mainly be due to the caffeine content of the extracts) (5), while other studies attribute beneficial effects only to the catechins in green tea (6, 7).

There are indications that thermogenesis induced by such green tea compounds in humans may be ascribed to BAT activity. The originally observed increase in energy expenditure (4) was paralleled by an increase in catecholamines in the urine and by a lowered RQ value, thus being compatible with increased sympathetic stimulation of lipolysis and thermogenesis in BAT. A BAT site of this thermogenesis is further implied by an observation that thermogenic effects of green tea compounds are only found in BAT-positive individuals (8).

For a more stringent analysis of the thermogenesis observed in connection with an intake of green tea compounds, studies in experimental animals are necessary. However, there is seemingly only one study that demonstrates an acute thermogenic effect of green tea compounds in mice: acute gavage of green tea compounds induced elevation of energy expenditure over a 5-h period following the gavage (i.e., longer than a stress effect of the gavage) (9). In indirect studies, increased energy expenditure in rats fed green tea compounds has been calculated from food intake and body energy content data (10). These studies, thus, imply that thermogenesis induced by green tea compounds may also be found in experimental animals. There is presently no direct evidence that the thermogenesis induced by green tea compounds in mice and rats originates from BAT activity; there are, e.g., no examinations to date of the existence or not of such thermogenesis in UCP1-ablated mice.

However, chronic stimulation of BAT thermogenic activity is generally associated with recruitment of the tissue: an increased thermogenic capacity. To the degree that such recruitment can be observed in animals chronically treated with green tea compounds, it may be considered indirect evidence that BAT activity may mediate (some of) the thermogenic effect of green tea compounds. Evidence for such recruitment indeed exists: Chronic exposure to green tea compounds increases BAT total protein content (10), total BAT mitochondrial DNA content (11), and UCP1 expression (12), and such exposure also induces browning of WAT in diet-induced obese mice (13). In mice given green tea compounds chronically and acutely exposed to cold, increased heat emission could be detected by infrared thermography in the region of the interscapular BAT (14). There is, thus, good evidence for the recruitment of BAT by green tea compounds in intact animals.

The mechanism behind this recruitment has not been established. It could be due to enhanced stimulation of the tissue *via* the central nervous system and activation of sympathetic innervation, or due to direct effects on the adipocytes. Green tea compounds could activate controlling centers in the brain or they could enhance the effect of sympathetic stimulation. Catechin polyphenols from green tea can inhibit catechol-O-methyl-transferase (COMT) that catalyzes norepinephrine degradation (15, 16). In this way, a given adrenergic signal would last longer and lead to more stimulation of the tissue and possibly to more recruitment [although this mechanism is controversial (7)].

Alternatively, the recruitment of BAT by green tea compounds could be a cell-autonomous effect. This would mean that green tea compounds directly affect brown adipocytes *in situ* to enhance UCP1 gene expression and other thermogenic features; similarly, these compounds could directly promote the browning of certain white adipocytes. Despite the interest in the thermogenic effect of green tea compounds, such possibilities have not been studied in brown adipocytes. Regarding white adipocytes, there are studies in certain white adipocyte-like cell lines. In the mouse adipocyte-like cell line 3T3-L1, green tea compounds increased UCP1 expression (17). In a different mouse adipocyte-like cell line (3T3-F442A), green tea compounds led to enhanced potential for thermogenesis (18). Although these cell lines (3T3-L1 and 3T3-F442A) can accumulate lipids in triglyceride droplets and, thus, may be said to undergo adipose conversion, their cellular origin is unclear, and they may not be representative for brown adipocytes or for subcutaneous white adipocytes (i.e., those white adipocytes that physiologically can “brown”). It is, therefore, essential to examine the ability of green tea compounds to induce thermogenic recruitment in authentic brown and white adipocytes. Examination of such cell-autonomous effects of green tea compounds in these cells is, thus, the aim of this study.

## Materials and Methods

### Animals

The experiments were approved by the Animal Ethics Committee of the North Stockholm region (N 73/16). Male Naval Medical Research Institute (NMRI) outbred mice 3–4 weeks of age (purchased from Charles River Laboratories) were used for the preparation of primary cultures of brown and white adipocytes. The mice were held in the animal facility of Stockholm University for at least 24 h at room temperature on a 12:12-h light-dark cycle with free access to chow food (Labfor R70; Lantmännen, Södertälje, Sweden) and water. The mice were then euthanized with CO_2_, followed by cervical dislocation. All animal experiments were carried out in accordance with the U.K. Animals (Scientific Procedures) and EU Directive 2010/63/EU for animal experiments. Primary brown cultures were prepared from the pooled interscapular, cervical, and axillary BAT. Inguinal WAT depots were used to prepare primary white cultures. The entire depots were taken. Depots (brown and white) from eight mice per experiment were pooled and digested as described below. At least five independent cultures performed in duplicate were used for each experimental setup.

### Primary Cell Culture Conditions

The pooled tissue pieces were minced in DMEM (Sigma-Aldrich, D6429) and transferred to a digestion solution with 0.2 % (wt/vol) collagenase (type II; Gibco, 9001-12-1) in a buffer consisting of 0.1 M HEPES (pH 7.4), 123 mM NaCl, 5 mM KCl, 1 mM CaCl_2_, 4.5 mM glucose, and 1.5% (wt/vol) BSA. The digestion was performed for 30 min at 37°C in a shaking water bath with vortex mixing every 15 min. The cell suspension was filtered through a 250-μm pore-size nylon filter (Sintab, Oxie, Sweden) into sterile 15-ml tubes. The filtered suspension was kept on ice for 20 min to let the mature adipocytes float up. The top layer of the suspension was removed, and the rest of the suspension was filtered through a 25-μm pore-size nylon filter (Sintab) and centrifuged at 1,300 g for 13 min, to pellet preadipocytes. The pellet was resuspended in 10 ml of DMEM and centrifuged at 1,300 g for 13 min. The pellet was then suspended in culture medium (0.2 ml/animal).

The cells were cultured in Corning 12-well plates containing 1.0 ml of culture medium added to each well before 0.1 ml of cell suspension was added. The culture medium was DMEM with 10% (vol/vol) newborn calf serum (Sigma-Aldrich, N4637), 2.4 nM insulin (Sigma-Aldrich, I9278), 25 μg/ml sodium ascorbate (Sigma-Aldrich, A4034), 10 mM HEPES (Sigma-Aldrich, H0887), 4 mM glutamine (Sigma-Aldrich, G7513), 50 U/ml penicillin, and 50 μg/ml streptomycin (Sigma-Aldrich, P0781). Seeding is considered day 0, and the culture medium was changed 24 h after seeding and every 48 h thereafter. The new medium was prewarmed to 37°C before being used. The medium was not changed on the day the cells were harvested (19). Cells were treated with green tea extracts (see below) or with four catechins mixed at the time of medium changes and supplemented or not (when indicated) with 1 or 0.1 μM rosiglitazone maleate (Alexis Biochemicals) dissolved in ethanol (ethanol final concentration 0.5%). The cells were grown at 37°C in an atmosphere of 8% CO_2_ in the air with 80% humidity. After 7 or 9 days (as indicated), cells were treated for 2 h with water (control) or with 1 μM norepinephrine (Sigma-Aldrich, A9512). Cells were harvested in Tri Reagent (Sigma-Aldrich, T9424).

### Green Tea Extract and Catechin Treatment

#### Green Tea Extract

A green tea water-soluble powdered extract (GTE) was purchased from Florien Fitoativos Ltda (Piracicaba, São Paulo, Brazil). The green tea powder was stored in the refrigerator as powder, and on the day of the experiments, it was weighed (1 mg/ml), solubilized in DMEM, and freshly used. The concentrations of catechins in the extract were determined by HPLC. Total phenolic content (TPC) and flavonoid content were determined using Folin-Ciocalteu's phenol reagent and gallic acid (99% purity, Sigma), with the bioflavonoid rutin as a reference, as previously described (20). The concentration of polyphenols, catechins, and flavonoids in the extract was 52, 28, and 11%, respectively, while the content of caffeine in the extract was 3.4%. The standard dose of GTE used was 1.9 μg/ml, indicated as GTE. In some experiments, double this dose (2·GTE) or other doses were used, as indicated.

#### Catechin Treatment

Cells were treated with a mix of four catechins at a final concentration of 4 μM [epigallocatechin-3-gallate (EGCG) 2 μM, epigallocatechin (EGC) 1 μM, epicatechin gallate (ECG) 600 nM, and epicatechin (EC) 400 nM]. The concentration of catechins used in this study was similar to the proportion of catechins found in the GTE and to that previously used (18, 21). We will refer to the GTE and the catechins collectively as the “green tea compounds.”

Primary brown and white preadipocytes were treated with either the GTE or catechins during all 7 days of differentiation *or* for 48 h from day 7 to day 9, as indicated. After the period of differentiation, cells were collected for mRNA expression analysis.

### Isolation of RNA, Complementary DNA Synthesis, and Real-Time qPCR

Freshly harvested cells were homogenized in Tri Reagent. RNA was extracted using the chloroform-isopropanol method according to the instructions of the manufacturer. RNA (500 ng) was reverse-transcribed using the High Capacity cDNA Kit (Life Technologies, no. 4368814) in a total volume of 20 μl. Gene-specific primers were premixed with 11 μl SYBR Green JumpStart Taq Ready Mix (Sigma-Aldrich, S4438) to a final concentration of 0.3 μM. cDNA was diluted 1:10, and aliquots of 2 μl per reaction were run in triplicate. Thermal cycling conditions were 2 min at 50°C, 10 min at 95°C, and 40 cycles of 15 s at 95°C and 1 min at 60°C, followed by melting curve analysis on a Bio-Rad CFX Connect Real-Time system. The ΔCt method (2^−ΔΔCt^) was used to calculate the relative changes in mRNA abundance [i.e., Ct-values for transcription factor IIB (TFIIB) were subtracted from the Ct-value of each gene to adjust for any variability in cDNA synthesis]. Primer sequences are listed in Table 1. None of the variations observed in TFIIB expression affected any of the conclusions. TFIIB expression data are presented in each figure.

**Table 1:** Nucleotide sequences of primers used for RT-qPCR amplification.

**Gene (*Mus Musculus*)**	**Primer sense (5^**′**^-3^**′**^)**	**Primer anti-sense (3^**′**^-5^**′**^)**
TFIIB	TGGAGATTTGTC	GAATTGCCAAACTCA
	CACCATGA	TCAAAACT
Cd36	CGCACATTGAGATTCTTTTCC	TCCTTTAA5GGTCGATTTCAGATC
Fabp4/aP2	CGCAGACGACAGGAAGGT	TTCCATCCCACTTCTGCAC
Pparg	GAAAGACAACGGACAAATCACC	GGGGGTGATATGTTTGAACTTG
Ppargc1a	GAAAGGGCCAAACAGAGAGA	GTAAATCACACGGCGCTCTT
Cidea	GCCTGCAGGAACTTATCAGC	GCCTGCAGGAACTTATCAGC
Fgf21	AGATGGAGCTCTCTATGGATCG	GGGCTTCAGACTGGTACACAT
Ucp1	GGCCTCTACGACTCAGTCCA	TAAGCCGGCTGAGATCTTGT
Plin1	CTGAGGAGAACGTGCTCAGA	AGAGTGTTCTGCACGGTGTG
Dgat2	GGCGCTACTTCCGAGACTAC	TGGTCAGCAGGTTGTGTGTC
Fasn	AGTGTTCGTTCCTCGGAGTG	GCTGCTGTTGGAAGTCAGC

*Transcription factor II B (TFIIB), fatty acid translocase (Cd36), fatty acid-binding protein 4/adipocyte protein 2 (Fabp4/aP2), peroxisome proliferator-activated receptor gamma (Pparg), peroxisome proliferator-activated receptor, gamma, coactivator 1 alpha (Ppargc1a), cidea (cell death-inducing DNA fragmentation factor alpha-like effector A), uncoupling protein 1 (Ucp1), fatty acid synthase (Fasn), fibroblast growth factor 21 (Fgf21), perilipin 1 (Plin 1), diacylglycerol O-acyltransferase 2 (Dgat2)*.

### Oil Red O Quantification and Microscopy Analyses

Control and GTE- or catechin-treated, differentiated cells (day 7) were washed with PBS and fixed for 30 min in 4% paraformaldehyde. Cells were then washed with 60% isopropanol and stained with Oil Red O (Sigma-Aldrich, O0625) solution (0.5% w/v in isopropanol) for 30 min according to Reference (22). Cells were then washed with tap water until the wash-off was clear. The Oil Red O stain was extracted with 100% isopropanol (1,000 μl per well in a 12-well plate) for 10 min. The absorbance of the extracts was measured in an EnSpire® plate reader (Perkin-Elmer) at 510 nm. Values were corrected for the background signal and the signal obtained from Oil Red O extracted from an empty well in the cell culture plate. Before the Oil Red extraction, stained cells were analyzed by optical microscopy (Olympus SC30), and the images of each group were acquired using the software Analysis Five getIT.

### Statistical Analyses

All data points are mean values with SEs of the mean of at least five independent experiments (separate cell cultures). All cultures were performed in duplicate for each experimental group and analyzed independently. The data were analyzed by one-way ANOVA followed by Tukey's post-test. The software employed for statistical analysis was GraphPad Prism (GraphPad Software, San Diego, CA, United States).

## Results

### GTEs Do Not in Themselves Induce Thermogenic Recruitment in Adipocytes

To examine the possible existence of cell-autonomous effects of green tea compounds on the thermogenic capacity of brown and white adipocytes, we have exposed adipocytes, grown and differentiated in primary culture, to different green tea compounds, and examined the expression of thermogenesis-related genes. We have used a GTE at different doses and a mixture of defined polyphenols (catechins), as detailed in section Materials and methods.

To evaluate if green tea compounds can directly recruit thermogenic genes, brown and white preadipocytes were treated during their differentiation period with the GTE or catechins. Full differentiation of the primary preadipocytes has occurred 7 days after seeding (23). Therefore, chronic treatment for these 7 days was performed to evaluate the effect of the green tea compounds.

**Figure 1:**
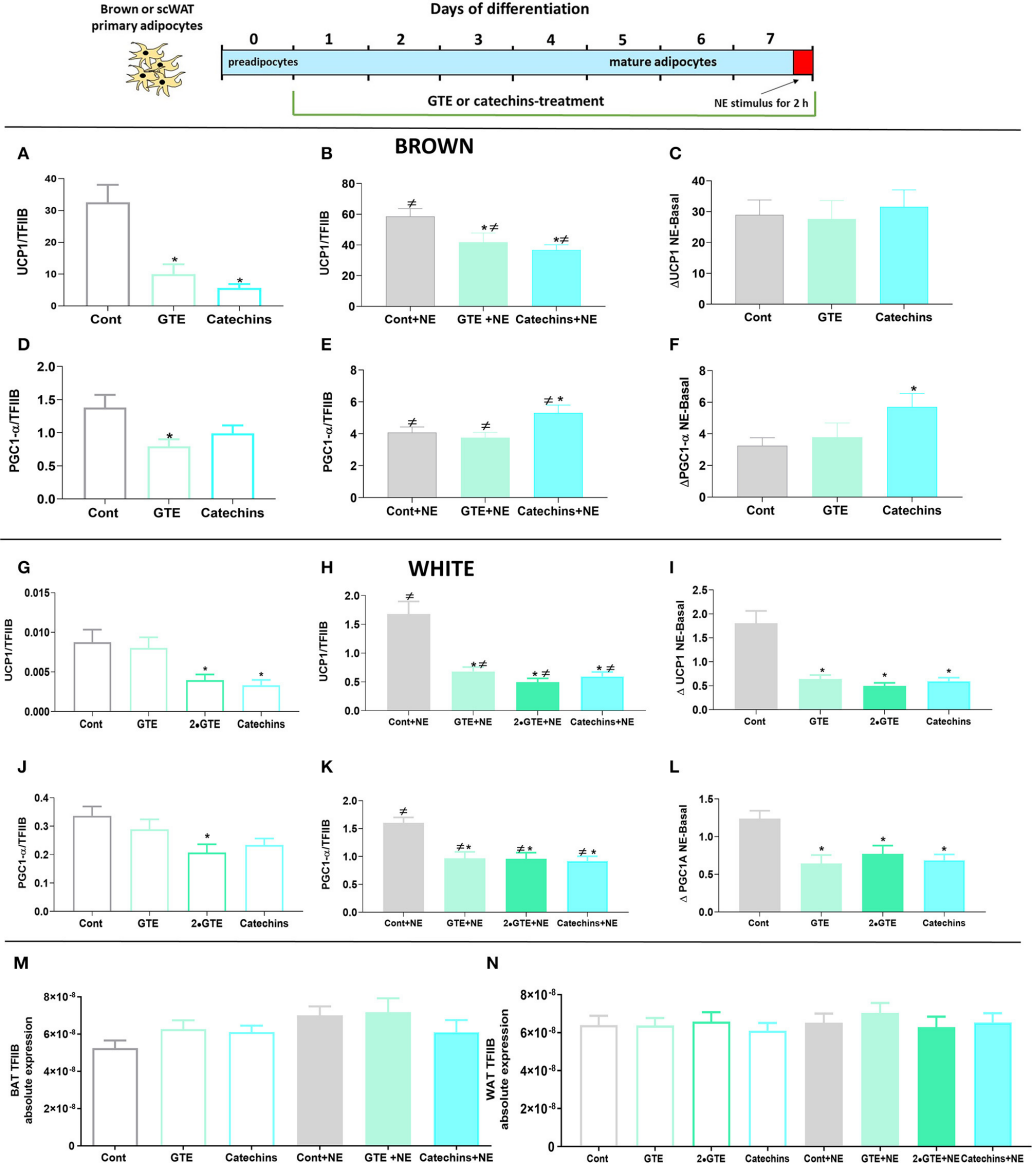
Effect of chronic treatment with green tea compounds on expression of thermogenic genes in primary adipocytes. Primary brown and white adipocytes were treated for 7 days with green tea extract (GTE, 1.9 and 3.8 μg/ml, 2•GTE) or catechins (4 μM). Two hours before harvesting, 1 μM norepinephrine (NE) was added to the cell cultures. **(A–C)** Brown adipocyte Ucp1 mRNA levels: basal **(A)**, norepinephrine (NE)-stimulated **(B)**, and the increase due to norepinephrine (**B** minus **A**) **(C)**. **(D–F)** Brown adipocyte Pgc1α mRNA levels, as in ABC. **(G–L)** Corresponding data for white adipocytes. Ucp1 and Pgc1α mRNA levels were expressed relative to the TFIIB levels in each sample (**M**: TFIIB mRNA levels in brown and **N** in white adipocytes). The results are means ± SEM of five independent experiments, each performed in duplicate. Statistical differences were determined by one-way ANOVA and Tukey's post-test. *p < 0.05 compared with the respective control group. ≠ *p* < 0.05 compared with the respective basal group. For gene abbreviations, as shown in Table 1.

There was a low level of UCP1 gene expression in untreated brown adipocytes (Figure 1A). The presence of either GTE or catechins during the differentiation period significantly reduced the already low UCP1 mRNA levels. These green tea compounds were, thus, in themselves not able to increase UCP1 gene expression during the differentiation process in cultured primary brown adipocytes.

In brown adipocytes, the activation of adrenergic receptors by norepinephrine triggers a signal transduction cascade that induces the expression of UCP1 (24). As therefore expected, norepinephrine stimulation (for the final 2 h) led to a clear increase in UCP1 mRNA levels. However, also in the norepinephrine-stimulated cells, the final level of UCP1 mRNA was lower in the GTE-treated, and the catechin-treated brown adipocytes than in the untreated control cells (Figure 1B). There was, thus, no positive interaction between the green tea compounds and adrenergic stimulation.

To delineate whether the green tea compounds affected the magnitude of the norepinephrine-induced response as such, we subtracted the UCP1 mRNA level in the cell cultures that had not been stimulated with norepinephrine from the levels of the norepinephrine-stimulated cells, thus obtaining the response to norepinephrine as such. As shown in Figure 1C, the norepinephrine response was identical in the presence or absence of the green tea compounds. As the effect of norepinephrine, thus, starts from a lower level, the relative effect of norepinephrine appears to be higher after treatment with the green tea compounds. However, this would be a misrepresentative interpretation of the results, as the absolute effect of norepinephrine is the same. Thus, the green tea compounds do not affect the response to the norepinephrine pathway for thermogenic recruitment, and effects of these substances would seem to occur through the independent mechanisms.

In the white adipocytes, the basal expression level of UCP1 was even much lower (about 3,000-fold lower) than in the brown adipocytes. The green tea compounds decreased basal UCP1 expression in the white adipocytes, as they did in the brown adipocytes (Figure 1G). In the white control adipocytes, norepinephrine stimulation led to a very markedly relative increase in UCP1 gene expression (about 200-fold), although the level was still 50 times lower than in the brown adipocytes. The green tea compounds lowered the UCP1 mRNA levels also in the norepinephrine-stimulated white cells (Figure 1H). In these cells, this included an inhibitory effect of the green tea compounds on the norepinephrine-induced increase in UCP1 expression (Figure 1I).

We also followed the expression of the transcriptional coactivator controlling mitochondrial recruitment: PGC1α. Under basal conditions, brown adipocytes treated with both of the green tea compounds tended to show reduced levels of PGC1α (Figure 1D). After norepinephrine stimulation, PGC1α levels were increased in the presence of catechins (but not of GTE) (Figure 1E). The effect of norepinephrine on PGC1α mRNA levels was augmented by catechins; there was, thus, a synergistic interaction between norepinephrine and catechins (Figure 1F). In white adipocytes, the green tea compounds reduced PGC1α mRNA in themselves (Figure 1J), and the green tea compounds also lowered the norepinephrine effect (Figures 1K,L). Thus, we found no evidence that green tea compounds could cell-autonomously induce thermogenic recruitment in either brown or white adipocytes during the differentiation process.

### Green Tea Compounds Lower Lipid Accumulation

**Figure 2:**
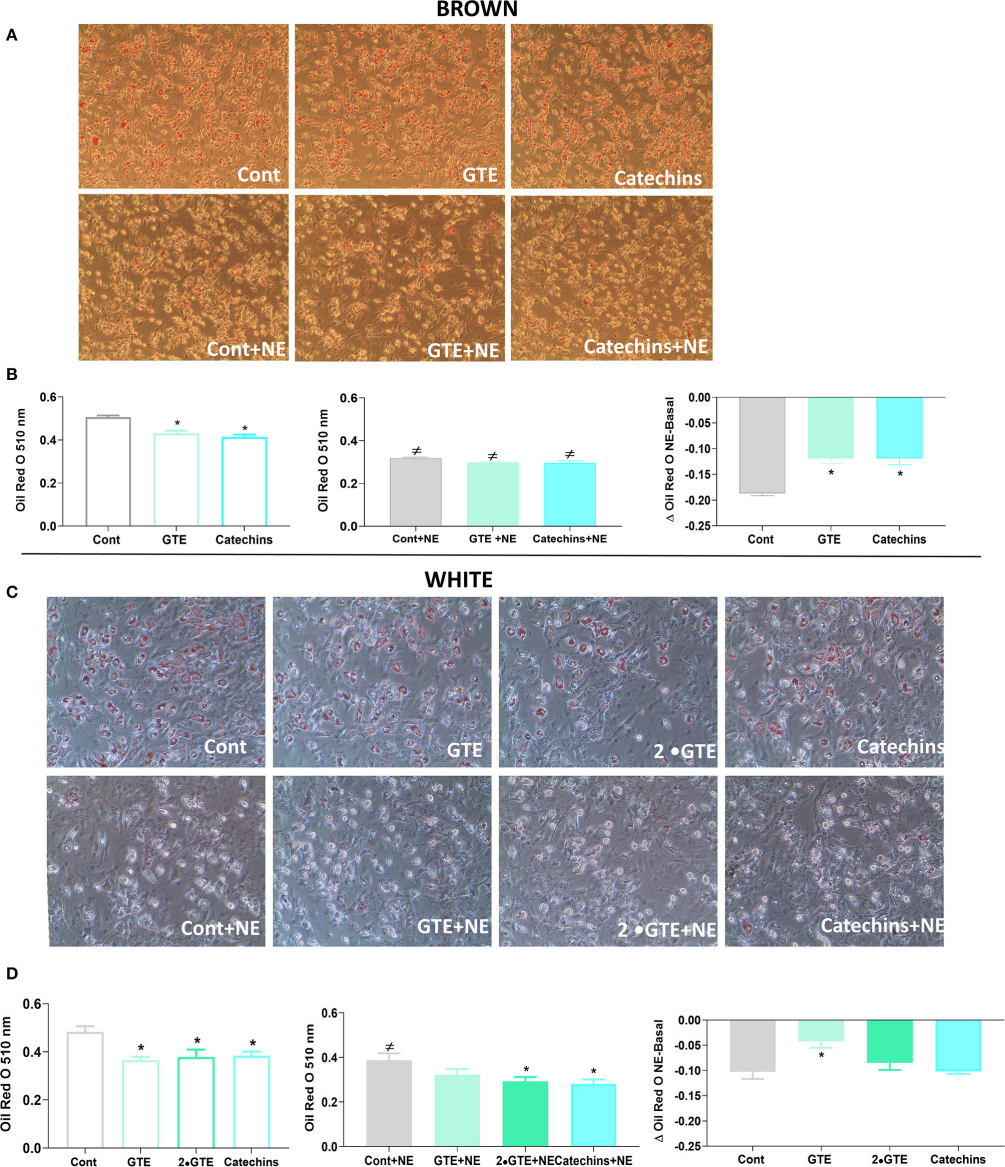
Effect of green tea compounds on lipid accumulation. Representative morphology of primary brown **(A)** and white adipocytes **(C)** is described in Figure 1. After norepinephrine stimulation, the adipocytes were stained with Oil Red O as described in section Materials and methods. Quantification of Oil Red O staining of brown **(B)** and white adipocytes **(D)** in basal and NE-stimulated states and the norepinephrine-induced change. The results are means ± SEM of four independent experiments, each performed in duplicate wells per group. Statistics are as in Figure 1.

To evaluate the general differentiation state in the adipocytes treated with the green tea compounds, we followed the lipid accumulation by Oil Red O staining. Representative images from cell cultures of each group are shown in Figure 2A. To quantitate the lipid amounts in the cells, the Oil Red O levels were determined (Figure 2B). In brown adipocytes, the green tea compounds in themselves reduced lipid accumulation. Norepinephrine, as expected, lowered lipid amounts [due to induced lipolysis (25)], but this effect was lower in the cells treated with green tea compounds (Figure 2B).

Representative images of white adipocytes stained with Oil Red O from each group are presented in Figure 2C. In these adipocytes, the green tea compounds themselves also reduced lipid accumulation. The effect of norepinephrine itself was not systematically affected by the green tea compounds (Figure 2D).

### Expression of Lipogenic Genes Is Altered by Green Tea Compounds

**Figure 3:**
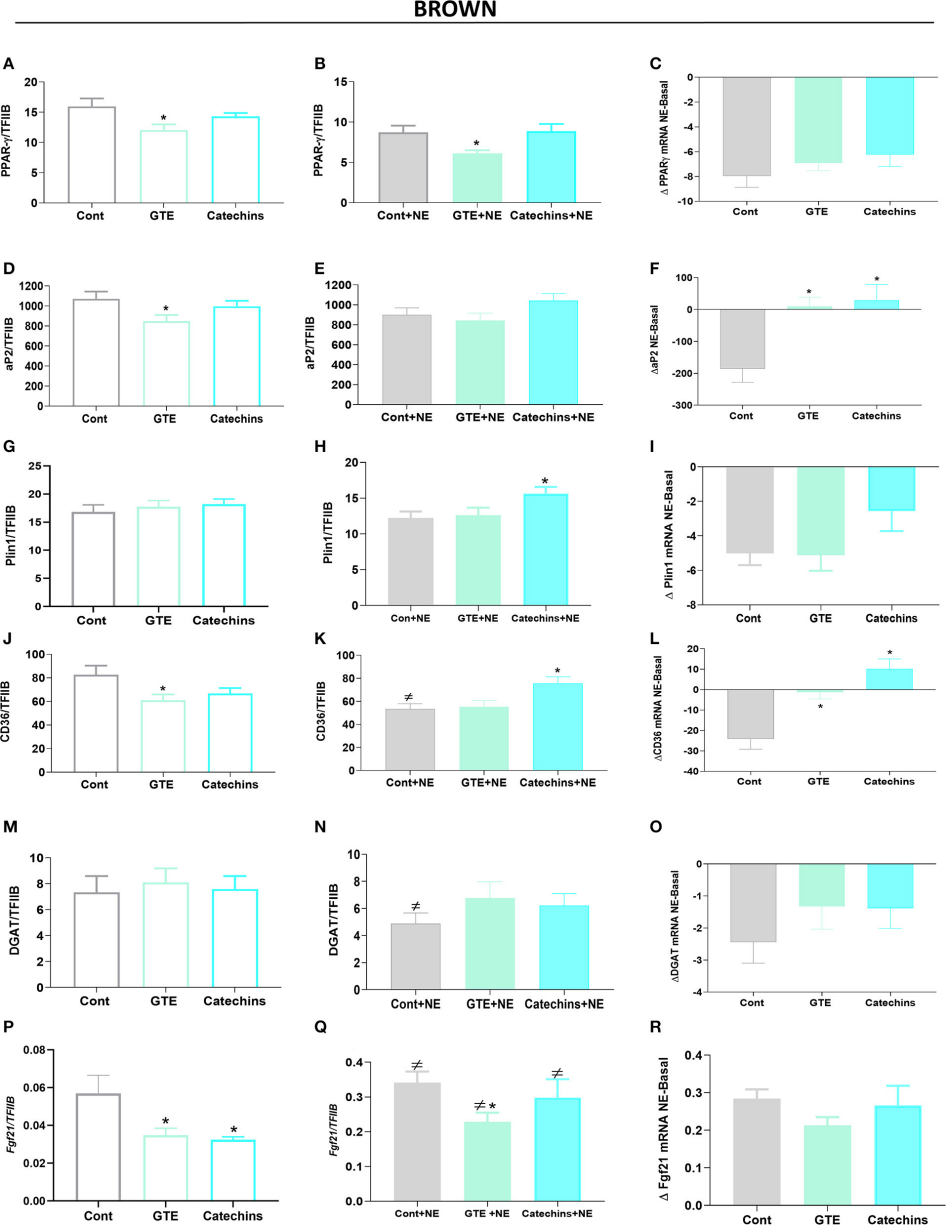
Effect of green tea compounds on expression of genes associated with lipid metabolism in brown adipocytes. The brown adipocytes in Figures 1, 2 were further analyzed for the expression levels of genes associated with lipid metabolism. *PPAR*γ mRNA levels in basal **(A)** and NE-stimulated **(B)** states, and the norepinephrine-induced change **(C)**. **(D–F)** results for *aP2*; **(G–I)** results for *Plin1*; **(J–L)** results for *CD36*; **(M–O)** results for *DGAT*; **(P–R)** results for *Fgf21*. The results are means ± SEM of five independent experiments, each performed in duplicate. Statistics are as in Figure 1.

Since lipid accumulation was reduced in both brown and white adipocytes after treatment with green tea compounds, we measured the mRNA levels of several genes involved in lipogenesis: PPARγ, fatty acid-binding protein 4 (FABP4, aP2), perilipin (Plin1), the fatty acid transporter CD36, diglyceride acyl CoA transferase (DGAT), and fibroblast growth factor 21 (FGF21). In general, the expression of these genes in brown adipocytes tended to be slightly reduced by the green tea compounds (Figures 3A,D,J,P); Plin 1 and DGAT were not modified by the treatments (Figures 3G,M–O).

Norepinephrine stimulation in itself generally slightly decreased the expression of all these genes (Figures 3B,E,H,K,N,Q), and this was particularly evident when the norepinephrine effect in itself was calculated (Figures 3C,F,I,L,O,R). Exposure of the cells to green tea compounds during the differentiation period generally reduced the ability of acute norepinephrine treatment to decrease the expression of these genes.

**Figure 4:**
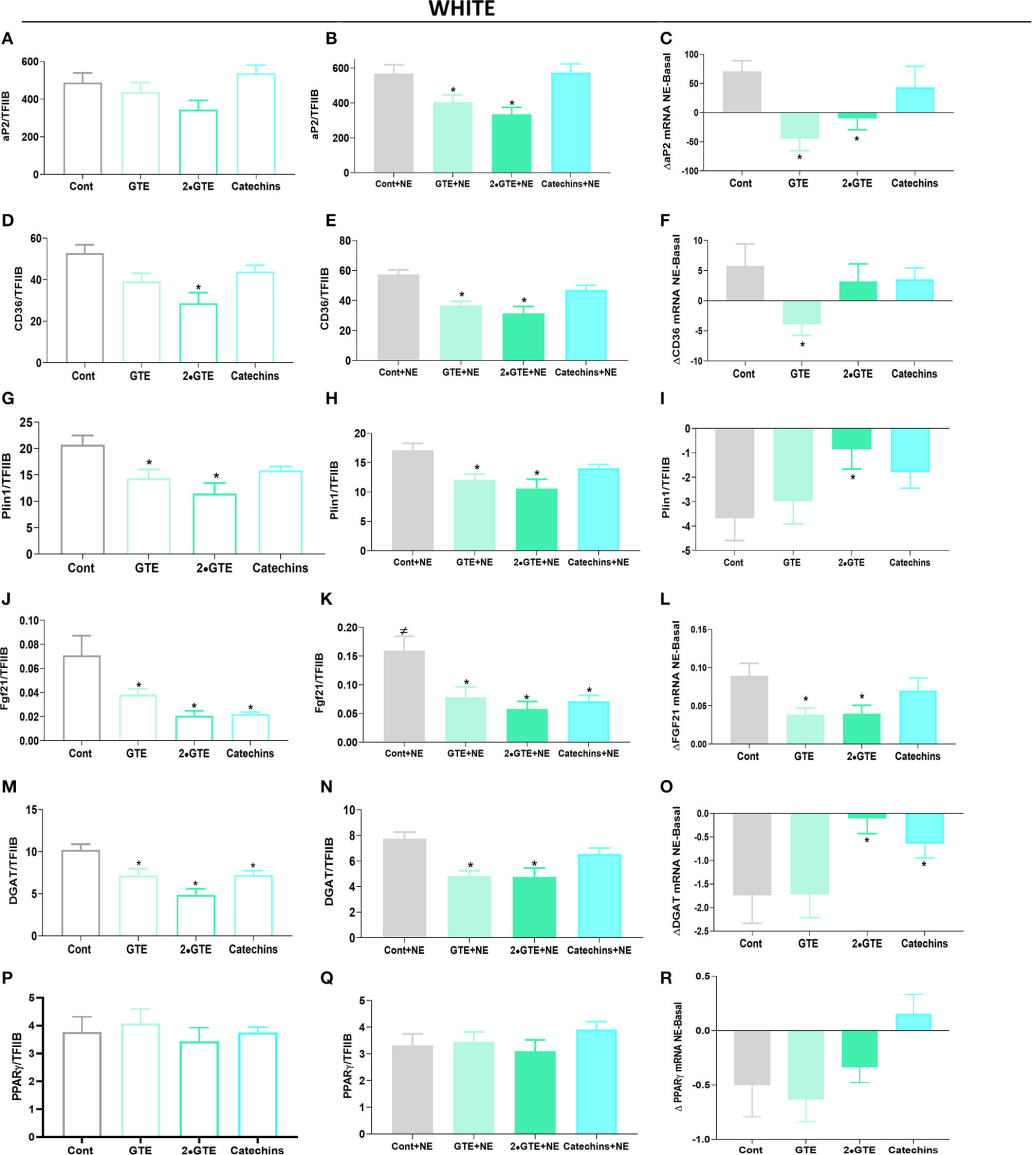
Effect of green tea compounds on expression of genes associated with lipid metabolism in white adipocytes. The white adipocytes in Figures 1, 2 were further analyzed for the expression levels of genes associated with lipid metabolism. *aP2* mRNA levels in basal **(A)** and NE-stimulated states **(B)** and the norepinephrine-induced change **(C)**. **(D–F)** results for CD36; **(G–I)** results for Plin1; **(J–L)** results for Fgf21; **(M–O)** results for DGAT; **(P–R)** results for PPARγ. The results are means ± SEM of five independent experiments, each performed in duplicate. Statistics are as in Figure 1.

In white adipocytes treated with the green tea compounds, the expression of the genes selected for analysis was generally reduced (Figures 4A,D,G,J,M) or not modified (PPARγ) (Figure 4P). In these white adipocytes, no significant suppressive effect of norepinephrine was evident (Figures 4B,E,H,K,N,Q), in contrast to what was the case in the brown adipocytes. In the cells treated with green tea compounds in the presence of norepinephrine, the general reduction in expression was maintained (Figures 4B,C,E,F,H,I,K,L,N,O,R).

We, thus, conclude that the inhibitory effect of green tea compounds on thermogenic recruitment seen above (Figure 1) is concomitant with a modest general inhibition of cell adipogenic differentiation.

### In Differentiated Adipocytes, Green Tea Compounds Retain Their Inhibitory Effect

As the inhibitory effects of the green tea compounds appeared to be due to general suppression of differentiation, we designed an experimental outline to avoid these effects. In these experiments, the cell cultures were allowed to fully differentiate in the absence of the green tea compounds and were, thereafter, exposed to the compounds for 2 days.

**Figure 5:**
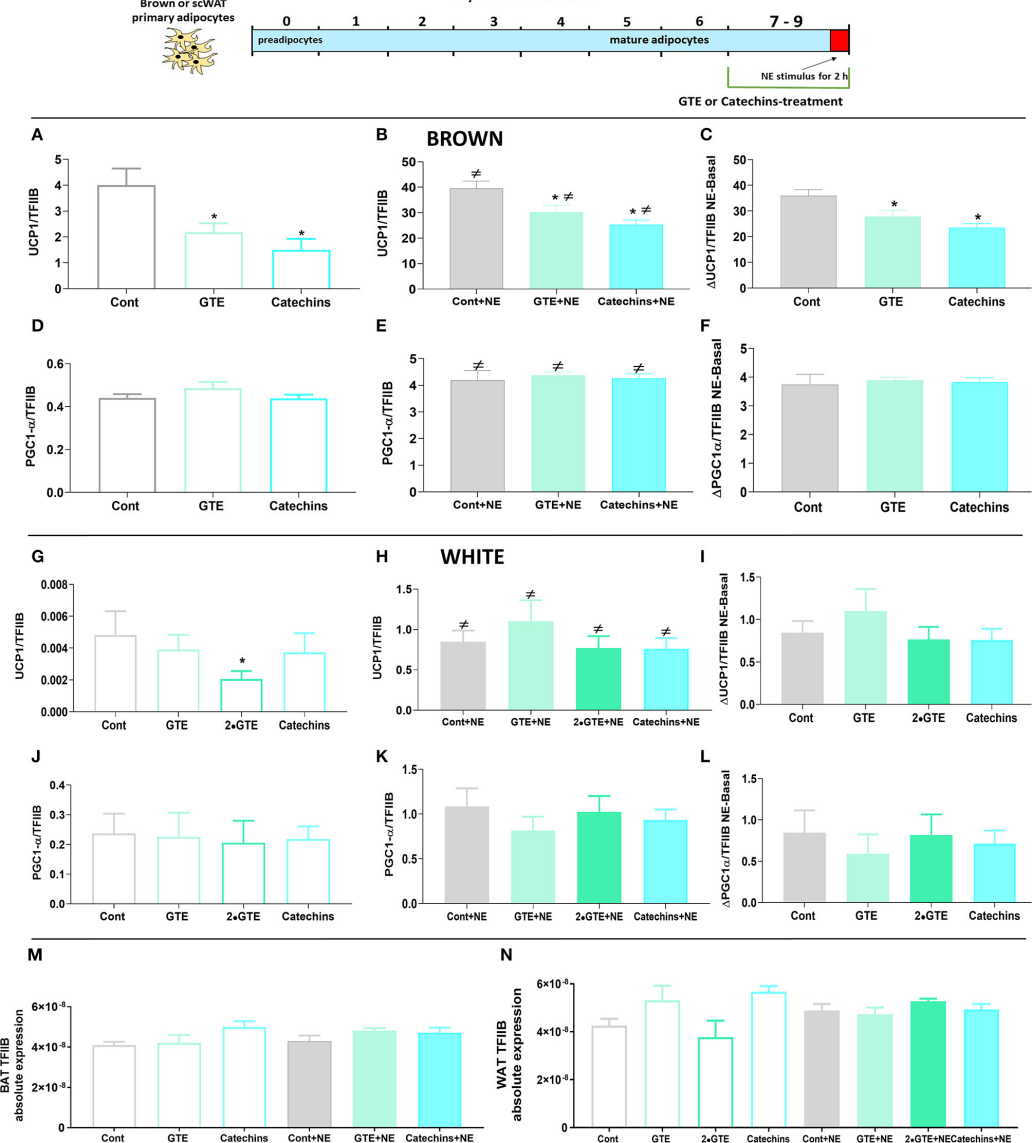
Effect of green tea compounds on mature brown and white adipocytes. Brown and white adipocytes were grown in culture for 7 days and then treated with green tea compounds (as specified in Figure 1) for 2 days and finally stimulated with norepinephrine for 2 h. Brown adipocyte Ucp1 mRNA levels in basal **(A)** and NE-stimulated states **(B)** and the norepinephrine-induced change **(C)** are shown. **(D–F)** results for Pgc1α. **(G–I)** results for UCP1 in white adipocytes; **(J–L)** results for Pgc1α in white adipocytes. Ucp1 and Pgc1α mRNA levels were normalized to the TFIIB levels in each sample (**M** TFIIB levels in brown and **N** in white adipocytes). The results are means ± SEM of five independent experiments, each performed in duplicate. Statistics are as in Figure 1.

However, also under these conditions, there was an observable inhibitory effect of the green tea compounds. Under basal conditions in the mature brown adipocytes, the green tea compounds decreased UCP1 gene expression (Figure 5A). Also, the UCP1 mRNA levels achieved after norepinephrine stimulation were lower in the presence of the green tea compounds (Figure 5B), and the absolute effect of norepinephrine in itself was lower in the presence of green tea compounds (Figure 5C). PGC1α expression levels were not affected by the green tea compounds under these conditions (Figures 5D–F). In mature white adipocytes, there were no consistent effects of green tea compounds on UCP1 or PGC1α gene expression (Figures 5G–L).

From these experiments, we conclude that while the green tea compounds, when added to mature brown or white adipocytes, generally did not inhibit the differentiated state that had been achieved, they did not induce UCP1 gene expression; rather, they diminished both the basal and the norepinephrine-induced expression levels. Thus, the cell-autonomous effects observed were not of a thermogenically enhancing nature.

### Chronic Treatment With Green Tea Compounds Reduces the Attainment of the Brite/Beige Adipocyte Phenotype

The thermogenic differentiation, “browning,” of brown adipocytes can be strongly induced by chronic treatment of the cell cultures with a PPARγ agonist such as rosiglitazone (26–28). Additionally, also certain white adipocytes are “browned” through rosiglitazone treatment (29–31). These cells are then referred to as brite (29) or beige (32) adipocytes. Similar to classical brown adipocytes, these brite/beige adipocytes express UCP1 at high levels, but in other respects, their gene expression profile is different from that of classical brown adipocytes and normal white adipocytes (29, 33). We, therefore, examined whether rosiglitazone treatment could overcome the negative effect of the green tea compounds on the differentiation process in white adipocytes (and in brown adipocytes), and whether there could be a positive interaction between rosiglitazone and the green tea compounds.

**Figure 6:**
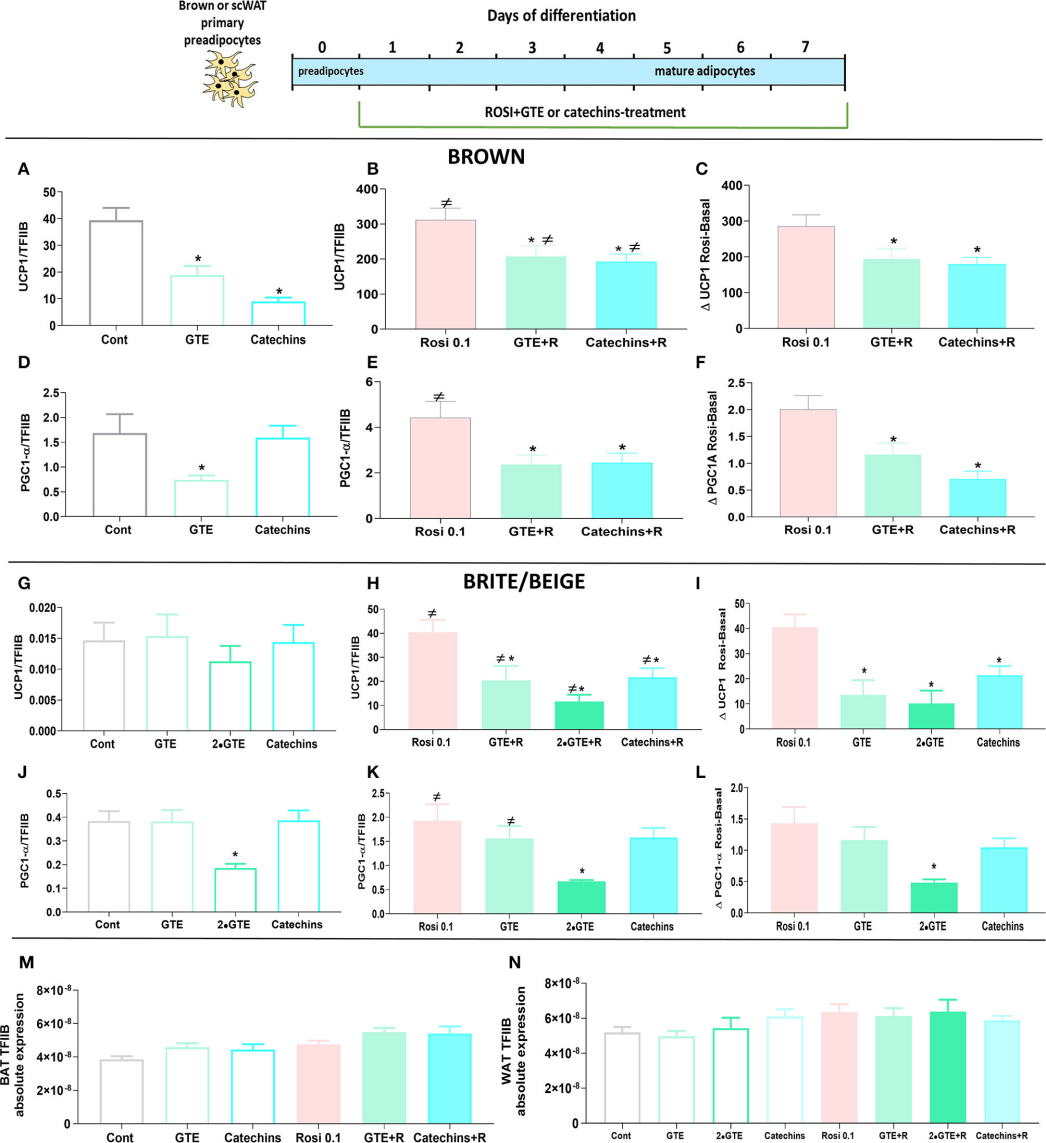
Effect of green tea compounds on rosiglitazone-induced differentiation. Brown and white primary adipocytes were treated with the green tea compounds specified in Figure 1 in the presence or absence of rosiglitazone (Rosi 0.1 μM). Brown adipocyte Ucp1 mRNA levels in basal **(A)** and rosiglitazone-stimulated states **(B)** and the rosiglitazone-induced change **(C)**. **(D–F)**: results for Pgc1α. **(G–I)** results for white adipocyte Ucp1; **(J–L)** results for white adipocyte Pgc1α. Ucp1 and Pgc1α mRNA levels were normalized to the TFIIB levels in each sample (**M** TFIIB levels in brown and **N** in white adipocytes). The results are means ± SEM of five independent experiments, each performed in duplicate. Statistics are as in Figure 1.

In mature brown adipocytes in the basal state, the green tea compounds again reduced the UCP1 level (Figure 6A), as before (Figure 1A). As expected (19, 29), the treatment of cells with rosiglitazone for 7 days increased the levels of UCP1 about 10-fold (Figure 6B); however, the presence of the green tea compounds during the rosiglitazone differentiation process reduced the level of UCP1 mRNA even in these cells (Figure 6B). The green tea compounds indeed lowered the effect of rosiglitazone as such (Figure 6C). Similar observations were made concerning PGC1α (Figures 6D–F).

In white adipocytes, rosiglitazone increased UCP1 gene expression 2,500-fold, to levels similar to those in untreated brown adipocytes, as expected (29). Thus, these adipocytes could be considered to have been induced to become brite/beige adipocytes. However, also in these brite/beige adipocytes, chronic treatment with green tea compounds lowered the ability of rosiglitazone to enhance UCP1 gene expression (Figures 6G–I). Also, PGC1α was increased some 5-fold by rosiglitazone treatment. The presence of GTE (especially in higher amounts) inhibited PGC1α gene expression (Figures 6J–L). We, thus, conclude that the presence of the differentiation inducer rosiglitazone could not overcome the negative effects of the green tea compounds on differentiation.

### Green Tea Extract Reduces Differentiation Even in the Presence of Rosiglitazone

To examine whether the green tea compounds acted on brown and white adipocytes by antagonizing the differentiation-promoting effects of PPARγ, we analyzed adipocytes after the promotion of differentiation. Cells were allowed to differentiate in the absence of rosiglitazone and were then treated for 2 further days with increasing concentrations of GTE in the absence or presence of rosiglitazone. If the GTE and rosiglitazone directly interacted negatively and positively, respectively, on the same site on PPARγ, we would expect to see a reduced ability of the GTE to inhibit UCP1 gene expression in the presence of rosiglitazone.

**Figure 7:**
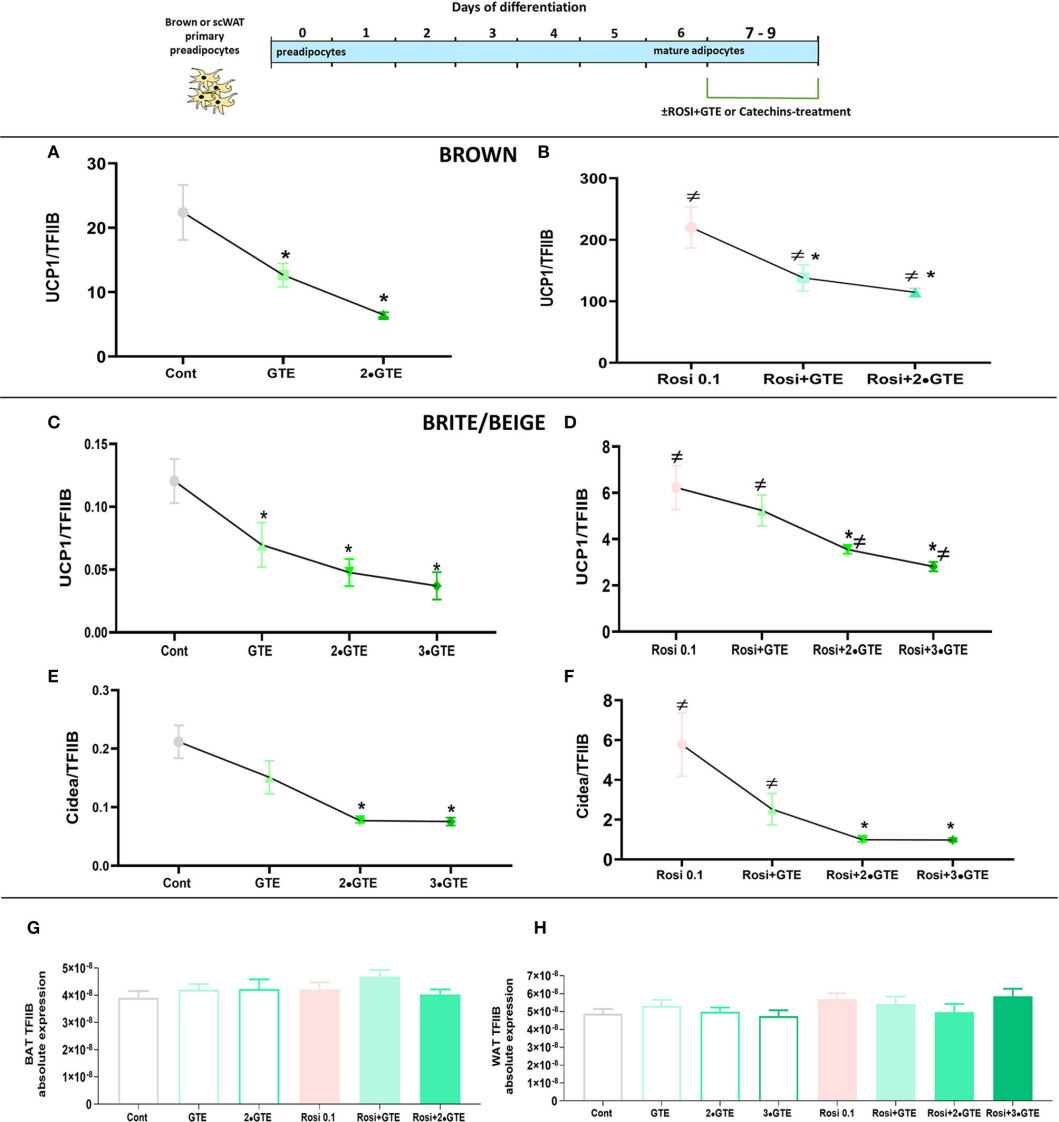
Interaction between green tea extract and rosiglitazone. Mature brown and white adipocytes were differentiated until day 7 and treated thereafter, from day 7 to day 9, with the indicated doses of green tea extract (GTE), in the presence or absence of rosiglitazone. Ucp1 mRNA levels in brown adipocytes in basal **(A)** and rosiglitazone-stimulated **(B)** cells. **(C,D)** results for UCP1 mRNA levels in white adipocytes. **(E,F)** results for Cidea mRNA levels in white adipocytes. Ucp1 and Cidea mRNA levels were normalized to the TFIIB levels in each sample (**G** TFIIB levels in brown adipocytes and **H** in white adipocytes). The results are means ± SEM of four independent experiments, each performed in duplicate. Statistics are as in Figure 1.

In untreated brown adipocytes, the GTE decreased UCP1 gene expression (Figure 7A), principally as seen above (Figure 5), in a dose-dependent manner. Treatment with rosiglitazone for 2 days again increased the level of UCP1 mRNA about 10-fold (Figure 7B). However, also in these rosiglitazone-treated cells, GTE reduced UCP1 mRNA levels in a dose-dependent manner (Figure 7B), to the same extent as in the rosiglitazone-untreated brown adipocytes.

Also in white adipocytes, the GTE reduced UCP1 levels dose dependently (Figures 7C,D), and the presence of rosiglitazone did not markedly shift the dose-response curve. Similar observations were made when another PPARγ target gene, Cidea (34), was followed (Figures 7E,F). We, therefore, conclude that GTE did not directly interact with the PPARγ pathway to decrease UCP1 expression.

## Discussion

The possibility that nutritional compounds may affect energy balance by activating thermogenic processes in brown and brite/beige adipose tissues has attracted much interest. In the present study, we have examined whether a cell-autonomous effect of green tea compounds on brown or brite/beige adipocytes exists. Particularly, we have examined whether the augmenting effects of certain green tea compounds on the thermogenic potential that has earlier been observed in certain adipocyte-like cell lines are maintained when examined in primary cultures of cells directly obtained from the corresponding adipose tissue depots in mice. We conclude that in this cellular environment, which may be considered a more biologically relevant environment, no augmenting effects of the green tea compounds on thermogenic potential were seen. To the extent that green tea compounds have beneficial effects on energy balance, it would, thus, seem that they act centrally, i.e., *via* the central nervous system, and not *via* a direct cell-autonomous effect on brown or white adipocytes. In a broader context, the difference between results obtained in adipogenic cell lines and those obtained here in authentic brown and white adipocytes points to limitations in using adipogenic cell lines in studies intended to serve as translational models for cell-autonomous effects.

### Green Tea Compounds Are Negative for Adipogenesis

We found that green tea compounds in general affected the differentiation process in brown and white adipocyte cultures negatively. Particularly, we found negative effects on the expression of genes related to lipid metabolism, a feature reflected in less lipid accumulation. These observations are not without precedence in studies in adipocyte-like cell cultures. Several studies have reported an inhibitory effect of GTEs or polyphenols from green tea on adipogenesis *in vitro* (17, 35). Kim et al. (17) found that different catechins and gallic acid inhibited the accumulation of intracellular lipids in the 3T3-L1 cell line. We have not established the mechanism for the differentiation-inhibitory effect of green tea compounds, but our results imply that it does not involve competition of these compounds with the binding site on PPARγ for rosiglitazone or endogenous rosiglitazone-like compounds.

It may be suggested that the negative effect of green tea compounds on the expression of genes related to thermogenic potential could simply result from the induction of a reduced degree of differentiation in general. However, in brown adipocyte cultures, agents that are negative for adipogenesis are not necessarily associated with negative thermogenic potential. Rather, a lowered adipogenic conversion may be associated with a cellular bifurcation toward enhanced thermogenic potential (36). However, this was not the case here, where parameters of adipose conversion, as well as thermogenic potential, were reduced in the presence of green tea compounds. Additionally, negative effects of green tea compounds were also observed when these substances were added to cells that were fully differentiated.

### Established Adipogenic Cell Lines May Not Reflect the Phenotype and Epigenetics of Primary Cells

In attempts to identify candidates for the activation of thermogenesis in adipocytes, adipocyte-like cell lines have frequently been used, particularly the 3T3-L1 and 3T3-F442a cell lines. These cell lines accumulate lipid droplets and, thus, fulfill basic requirements for studying adipose conversion. However, their phenotype and epigenetics may not correspond to those of native brown or white adipocytes; indeed, their origin is undefined in relation to tissues and depot. Their phenotype and epigenetic status may therefore not reflect that of adipocytes *in situ*.

When such cell types have been used to examine the ability of green tea compounds to induce the expression of UCP1 and other genes related to thermogenic potential, positive effects of these compounds have been reported (11, 17), i.e., results opposite to those presented herein primary cultures of both brown and white adipocytes. We are not aware of studies with green tea compounds in other established white adipocyte-like cell lines such as C3H10T1/2 or hMADS cells, and thus, we cannot say whether similar outcomes would be observed in all white adipocyte-like cell lines. We are also not aware of any studies of green tea compounds on brown adipocyte-like cell lines.

That the outcomes are different in cell lines vs. primary adipocytes may not be surprising. Except for the ability of the cell lines to undergo adipose conversion, there is little reason to expect that these cell lines reflect the general epigenetic status of white or brown adipocytes. Indeed, their ability to, e.g., express UCP1 is very low. In Table 2, we have compiled the values for relative UCP1 mRNA levels in an adipogenic cell line (3T3-F442a) compared to the levels observed here in cultured primary brown or white adipocytes. As seen, although the basal level of UCP1 gene expression is about the same in the cell line and the white adipocytes, the ability of norepinephrine to induce expression is 100- to 1,000-fold higher in the white adipocytes. In the brown adipocytes, the differences are even much pronounced, with the basal UCP1 mRNA levels being 1,000-fold higher than in the cell line, and the induced levels some 10,000-fold higher. We believe that these inherent differences reflect large epigenetic differences: that the control of UCP1 expression in white or brown adipocytes is, thus, fully different from that in the cell lines. We would consider that primary cultures of adipocytes better replicate the epigenetic status of adipocytes *in situ*. In consequence of this, the translational validity of studies utilizing adipocyte-like cell lines to evaluate the ability of, e.g., nutraceutical products to affect thermogenic potential may be limited. In principle, extrapolation of the present studies would be that results obtained with adipocyte-like cell lines are not good indicators of effects found in authentic adipocytes, a conclusion that has also recently been forwarded elsewhere (37).

**Table 2:** A comparison between an established adipogenic cell line and primary cultured white and brown adipocytes with respect to UCP1 mRNA levels.

	**Ct-values**			**Relative mRNA levels**		
	**3T3-F442a**	**Primary white**	**Primary brown**	**3T3-F442a**	**Primary white**	**Primary brown**
Control	31.1	30.9	18.9	1	1.1	4 705
NE/Iso	30.9	23.2	17.6	1.1	239	11 585
Rosi	30.5	18.5	16.1	1.1	6 208	32 768

*Left side of the table shows mean Ct-values from qPCR determinations of UCP1 mRNA levels in the different types of cultures. The 3T3-F442a levels are our earlier observations [see Bolin, et al. (9)]; the values are from cultures treated with norepinephrine or isoprenaline (NE/Iso) for 24 h. The values for primary white and brown adipocytes are from the present studies (data underlying Figures 1, 5–7). The right side of the table shows calculations of the relative mRNA levels that the Ct-values would represent, based on the assumption that each Ct-unit represents a doubling of the mRNA amounts. These values are expressed after setting the level in control 3T3-F442a cells to 1*.

## Conclusions

We conclude that there was a negative effect of the green tea compounds on basal UCP1 gene expression in both brown and white primary adipocytes, in contrast to the positive effects earlier reported from studies in adipogenic cell lines. We suggest that the epigenetic status of the adipogenic cell lines is fundamentally different from that of genuine brown and white adipocytes, reflected, e.g., in several-thousand-fold differences in UCP1 gene expression levels, and thus, we suggest that results obtained with adipogenic cell lines cannot unreservedly be extrapolated as being relevant for authentic brown and white adipocytes *in situ*.

## Data Availability Statement

The raw data supporting the conclusions of this article will be made available by the authors, without undue reservation.

## Ethics Statement

The animal study was reviewed and approved by Animal Ethics Committee of the North Stockholm region (N 73/16).

## Author Contributions

RO: designed the experiments, conducted experiments, performed data analysis and interpretation, and contributed to the manuscript drafting. NP: conducted experiments. BC and JN: performed data analysis and interpretation and wrote the manuscript. All authors contributed to the article and approved the submitted version.

## Conflict of Interest

The authors declare that the research was conducted in the absence of any commercial or financial relationships that could be construed as a potential conflict of interest.

## Publisher's Note

All claims expressed in this article are solely those of the authors and do not necessarily represent those of their affiliated organizations, or those of the publisher, the editors and the reviewers. Any product that may be evaluated in this article, or claim that may be made by its manufacturer, is not guaranteed or endorsed by the publisher.
